# Effects of Ceftriaxone on Oxidative Stress and Inflammation in a Rat Model of Chronic Cerebral Hypoperfusion

**DOI:** 10.3390/bs12080287

**Published:** 2022-08-14

**Authors:** Apsorn Sattayakhom, Kosin Kalarat, Thatdao Rakmak, Sompol Tapechum, Arnaud Monteil, Chuchard Punsawad, Sarawoot Palipoch, Phanit Koomhin

**Affiliations:** 1 School of Allied Health Sciences, Walailak University, Nakhonsithammarat 80160, Thailand; 2 Center of Excellence in Innovation on Essential Oil, Walailak University, Nakhonsithammarat 80160, Thailand; 3 School of Informatics, Walailak University, Nakhonsithammarat 80160, Thailand; 4 School of Liberal Arts, Walailak University, Nakhonsithammarat 80160, Thailand; 5 Department of Physiology, Faculty of Medicine Siriraj Hospital, Mahidol University, Bangkok 10700, Thailand; 6 Institutde Génomique Fonctionnelle, University of Montpellier, CNRS, INSERM, 34094 Montpellier, France; 7 School of Medicine, Walailak University, Nakhonsithammarat 80160, Thailand

**Keywords:** ceftriaxone, white matter damage, oxidative stress, inflammation

## Abstract

Ceftriaxone (CTX) exerts a neuroprotective effect by decreasing glutamate excitotoxicity. We further studied the underlying mechanisms and effects of CTX early post-treatment on behavior in a cerebral hypoperfusion rats. The rats’ common carotid arteries (2VO) were permanently ligated. CTX was treated after ischemia. Biochemical studies were performed to assess antioxidative stress and inflammation. Behavioral and histological studies were then tested on the ninth week after vessel ligation. The 2VO rats showed learning and memory deficits as well as working memory impairments without any motor weakness. The treatment with CTX was found to attenuate white matter damage, MDA production, and interleukin 1 beta and tumor necrosis factor alpha production, mainly in the hippocampal area. Moreover, CTX treatment could increase the expression of glia and the glial glutamate transporters, and the neuronal glutamate transporter. Taken together, our data indicate the neuroprotective mechanisms of CTX involving the upregulation of glutamate transporters’ expression. This increased expression contributes to a reduction in glutamate excitotoxicity and oxidative stress as well as pro-inflammatory cytokine production, thus resulting in the protection of neurons and tissue from further damage. The present study highlights the mechanism of the effect of CTX treatment and of the underlying ischemia-induced neuronal damage.

## 1. Introduction

Cerebral ischemia is a major cause of death globally, wherein cerebral blood flow disruption leads to a decrease in glucose and oxygen supply to the brain tissue [1]. This can occur either globally or focally. Global ischemia occurs when cerebral blood flow is reduced in the brain. A reduction in blood flow in a specific region of the brain is known as focal ischemia [2]. The severity depends on the degree and duration of ischemia. When the cerebral blood flow completely ceases, it leads to complete ischemia. Complete ischemia as observed in the ischemic core in focal ischemia or cardiac arrest, and induces neuronal death via the necrosis pathway. The brain area surrounding the ischemic core or during incomplete ischemic area can be preserved if the cerebral blood flow is recovered in an appropriate time period. Incomplete ischemia or hypoperfusion occurs when the global cerebral blood flow is partially reduced and the amount of blood is not high enough to maintain cerebral metabolic activities, resulting in injury to neurons and glia cells [3]. Many conditions and animal models, including hemorrhage, intracranial hypertension, two-vessel occlusion (2VO), and four-vessel occlusion (4VO), were used to study the pathogenesis and investigate neuroprotective substances in these promising areas of the brain. The middle cerebral artery occlusion and two-vessel occlusion models are frequently used for the study of focal and global ischemia, respectively. The 2VO model can induce a chronic reduction in cerebral blood flow, which then induces neuronal injury and a cognitive deficit. Thus, a 2VO rat model is appropriate for studying the pathophysiology and discovering the neuroprotective effects of a new drug. The knowledge gained from this model can also be extended to understand related mechanisms in the ischemic penumbra area of a focal ischemic model [4].

Several mechanisms underly ischemic neuronal injury, including glutamate excitotoxicity, oxidative stress, and inflammation. Glutamate excitotoxicity subsequently causes necrosis and apoptosis depending on the ischemic severity. Reperfusion injury induces the excessive formation of reactive radical oxide species (ROS), which leads to neuronal injury and death. Neuroinflammation activates the immune system, which impairs axonal regeneration due to immunoreactive cells and forms a glial scar [5,6,7]. Excitotoxicity has been the common center stage of cerebral ischemia research. Glutamate is a major neurotransmitter in the brain and spinal cord, and plays critical roles in neuronal communication, neuronal growth, neuronal survival, and maturation. During cerebral ischemia, the excessive release of glutamate from presynaptic sites into the synaptic cleft and surrounding sites causes the global stimulation of the glutamate receptor (NMDAR), which in turn leads to calcium influx, causing calcium-induced neuronal cell death. In the normal state, glutamatergic transmission is partly reduced by excitatory amino acid transporters (EAATs). EAATs are responsible for clearing glutamate by taking up synaptic glutamate into glial cells. Thus, the dysfunction or dysregulation of EAATs can lead to excessive glutamate-mediated toxicity during ischemia. Among the five mammalian EAAT isoforms, EAAT2 is the most abundant and is primarily responsible for glutamate homeostasis in the central nervous system. The CNS expresses not only EAAT2 but also EAAT1 and EAAT3, while the expression of EAAT5 is limited to the cerebellum and EAAT4 predominantly expresses in the retina [8,9]. It has been reported that the β-lactam antibiotic ceftriaxone (CTX) has a neuroprotective and anti-ischemic effect via the upregulation of glutamate transporters and acceleration of glutamate uptake, resulting in a reduction in glutamate excitotoxicity [10,11]. In an in vivo stroke model, CTX treatment could reduce the infarct volume and acute mortality after middle cerebral artery occlusion and attenuated neuronal injury [12,13]. Other studies revealed that CTX could enhance memory retrieval after hypobaric-hypoxia-induced memory impairment, and improve neuronal injury, as well as learning and memory impairment, after 2VO chronic cerebral hypoperfusion induction [12,14]. However, there is no report on the underlying mechanisms of this neuroprotection in this condition. To further the understanding of these mechanisms, we used a 2VO rat model of chronic cerebral hypoperfusion to investigate the effect of CTX treatment for glutamate excitotoxicity on neuroinflammation, oxidative stress, and functional impairment, with a focus on learning and memory performance. CTX showed neuroprotective effects and reduced oxidative stress and inflammation in this model. This underlying mechanism suggests the benefits of a combined strategy for chronic cerebral hypoperfusion condition.

## 2. Materials and Methods

### 2.1. Animals and Surgical Procedure

Young adult (8 weeks) male Sprague Dawley rats were used as the animal model in this study. The National Laboratory Animal Centre, Salaya, Nakhon Pathom provided the rats in this study. They were habituated for a week before being transferred to cages. They were then maintained in cages under a 12-h light/dark cycle. Three rats were allocated to each cage. The room temperature was maintained at 25 °C. The rats were allowed free access to food and water throughout the experiments. The experimental procedures were approved by the Walailak University Institutional Animal Care and Use Committee (WU-IACUC), Walailak University, Thailand (protocol number: WU 002/2014; approval date: 1 April 2014). Seventy-six young adult male Sprague Dawley rats aged around 8 weeks were used. The three groups of rats were divided based on common carotid ligation with different ligation durations: a sham-operated group (sham), a vehicle-treated common carotid artery-ligated group (2VO-Veh), and a ceftriaxone-treated common carotid artery ligated group (2VO-CTX); assessments took place on the first day and third day, and in the first week and ninth week. These time points were used for the biochemical study. Behavioral and histological studies were conducted the eighth and ninth week after vessel ligation, respectively.

The surgical procedure was conducted as previously described [14]. The rats were intramuscularly anesthetized with ketamine at 60 mg/kg and xylazine at 6 mg/kg. After anesthesia induction, the rats were cut at the ventral cervical region of the neck. The carotid sheath and the vagus nerve were gently separated from the right and left common carotid arteries. Then, the common carotid arteries were permanently ligated using silk suture. The sham-operated rats underwent the same procedure as the other group except for the artery ligation. Rats were allowed to fully recover and were returned to their cages. All surgical techniques were carried out under sterile protocol. The rat body temperatures were maintained using light.

### 2.2. Drug Administration and Experimental Protocol

**Figure 1:**
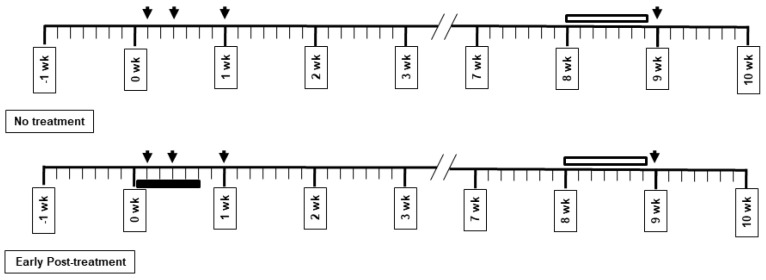
Experimental protocol of chronic cerebral hypoperfusion in rats. Arrow shows different sacrifice days. Dark block shows the duration of early post-treatment with ceftriaxone. Opened block shows the period of behavioral testing.

Ceftriaxone at a concentration of 25% (*w/v*) was diluted in sterile water (Siam Bheasach, Thailand). Ceftriaxone (200 mg/kg) treatment or vehicle was administered intraperitoneally once a day for 5 days. The treatment was started the first day after common carotid artery ligation. The tissue collection timeline is presented in Figure 1. The tissues were collected on the first day and third day, and in the first week and ninth week. The treatment was administered for 5 days after ischemic induction. Behavioral studies were performed in the eighth week after ischemic induction. Histological studies were performed in the ninth week, after the behavioral tests. Biochemical studies were performed on the first day and third day and in the first week and ninth week after vessel ligation.

### 2.3. Morris Water Maze Test

To test the success of ischemic induction, learning and working memory were tested using the water maze test after common carotid artery ligation at 8 weeks in the sham (*n* = 8) and 2VO-Veh (*n* = 8) groups. The training procedure was modified from the previous studies [15,16]. A circular pool of 200-cm diameter was used. A 25 cm water depth was prepared before testing. The transparent escape platform was submerged below the water surface and placed at the center of a dedicated zone. A digital video recorder was hung from the ceiling and directly connected to recording software in the adjacent room. The rats were trained for 120 s to find the hidden platform. Then, escape latencies were measured in four trials per day for 6 consecutive days. Spatial working memory was then tested after spatial acquisition. The platform was located in the same zone during the learning phase. After the learning trial, the platform was relocated to another quadrant of the water maze. The rats were allowed to test for 2 min in this trial [17], called a sample trial. The next consecutive trial was then performed with a 15 s inter-trial interval and was noted as the test or matching trial. The working memory was evaluated in the matching trial and also reported as escape latencies.

To investigate motor strength, a prehensile traction test was performed. Each rat’s forepaws were hung on a rope and then the hanging time was recorded as previously described [18]. Hanging times of 0, 0 to 2, and 3 to 4 s were scored as 0, 1, and 2, respectively. The scores were plotted as bar graphs to compare the motor strength between groups.

### 2.4. Histological Assessment

One day after behavioral testing, the rats were anesthetized with ketamine/xylazine and perfused with saline solution, followed by the transcardial perfusion of 4% paraformaldehyde (Unilab Chemicals, Ajax Finechem, New South Wales, Australia) in 0.1 M phosphate buffer (Unilab Chemicals, Ajax Finechem, New South Wales, Australia), pH 7.4. The rat brains were then removed and post-fixed in 4% paraformaldehyde solution at 4 °C for a day. The brains were processed using a tissue processor machine and then embedded in paraffin after the post-fixation method. Coronal sections were cut to a thickness of 10 μm. The coronal brain sections were mounted on slides. White matter damage was investigated using Luxol fast blue (Sigma-Aldrich, St Louis, MO, USA). The slides were recorded under a bright-field microscope. Pictures of the corpus collosum were taken at 400 times magnification. For each brain, a series of four sections of right and left brains were analyzed, with an interval of 200 μm. The severity of white matter damage was graded as previously described [19]. Scores of 0, 1, 2, and 3 were graded as normal, disarrangement of the axon fibers, vacuole formation, and the myelinated fiber disappearance, respectively. Immunohistochemistry was performed in hippocampal slides to evaluate gliosis and glutamate transporter expression. Glial fibrillary acidic protein (GFAP) (GFAP Ab; Santa Cruz biotechnology, Santa Cruz, CA, USA) was stained to count astrocyte number. The number of GFAP-positive cells was counted in three random fields at 200-time magnification (approximately 0.37 mm^2^). The OX-42 antibody (Santa Cruz biotechnology, Santa Cruz, CA, USA) for cluster of differentiation molecule 11b (CD11b) was used to investigate microglia. Excitatory amino acid transporters 1–3 (EAAT1-3) (Santa Cruz biotechnology, Santa Cruz, CA, USA) were assessed to measure glutamate transporter expression in the tissue. For OX-42 and EAAT1-3, the incubation times of horseradish peroxidase substrate were adjusted to be identical among slides to create an appropriate intensity with the same reaction activity. The modal gray values were then measured using ImageJ as previously described [20]. The immunoreactive signal intensity of OX-42 and EAAT1-3 was analyzed among groups.

### 2.5. Biochemical Measurement

The rats were divided into three groups for biochemical measurement and were sacrificed on the first day and third day and in the first week and ninth week after ischemic induction. The brain was dissected and isolated to collect the cortex, striatum, and hippocampus [21]. The brains were stored at −80 °C before use. The brains were then quickly homogenized with a handheld homogenizer on ice in NP40 cell lysis buffer (Sigma-Aldrich, St Louis, MO, USA). The brain homogenate was centrifuged for 10 min at 14,000× *g* and the supernatant was collected for protein measurement using the Bradford assay (Bio-Rad, Hercules, CA, USA). Malondialdehyde (MDA) was measured using a heating method as previously described [22]. The product of thiobarbituric acid reactive substances was spectrophotometrically measured at the same protein level. In total, 300 µL of sample and 700 µL of water were incubated at 37 °C for 30 min. Two milliliters of trichloroacetic acid (Sigma-Aldrich, St Louis, MO, USA) was added and boiled at 95 °C for 30 min, then cooled at room temperature. The mixture was centrifuged for 10 min at 1000× *g*. The supernatant was removed and measured at 532 nm. The levels of MDA were analyzed using standard curves and compared as a percent of the sham-operated control group. Pro-inflammatory cytokines were measured using enzyme-linked immunosorbent assay (ELISA) (PeproTech, London, UK). Interleukin 1 beta (IL-1β) and tumor necrosis factor alpha (TNF-α) were measured to evaluate neuroinflammation in the early and late stages of ischemia.

### 2.6. Data Analysis

Results are expressed as mean ± SEM. Normality was tested using Shapiro–Wilk test and all the presented data passed normality. The escape latencies from the Morris water maze test were analyzed using an unpaired *t*-test. Histological and biochemical data were analyzed using a one-way ANOVA. An unpaired *t*-test was used to evaluate the difference. Statistical significance was accepted at *p* < 0.05.

## 3. Results

### 3.1. Effects of Bilateral Common Carotid Artery Occlusion on Learning and Memory Performance

**Figure 2:**
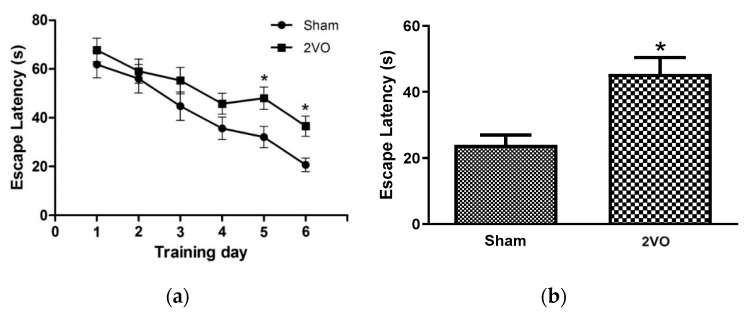

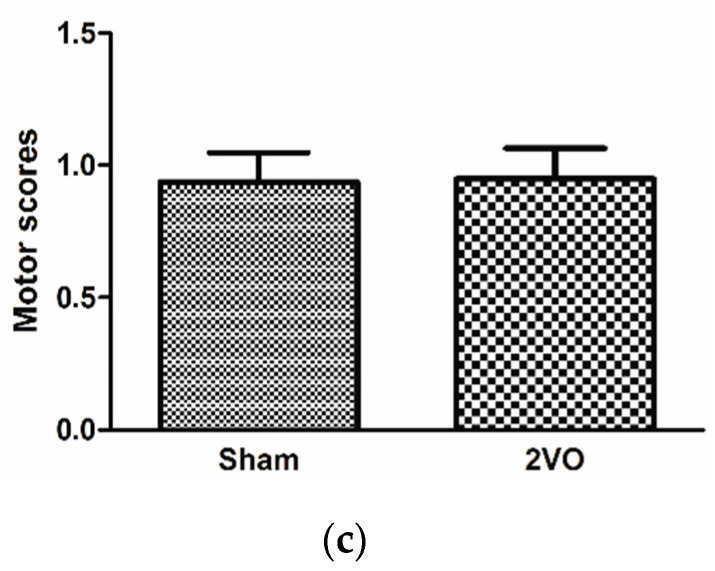
The Morris water maze test for rats in the 8th week. In the acquisition trial, escape latencies of sham-operated rats and 2VO rats were compared (**a**) and matching to the sample test was performed to test working memory (**b**). Prehensile traction test was performed and reported as motor scores (**c**). Data are expressed as mean ± SEM (*n* = 8). n.s. represents not statistically significant, * *p* < 0.05 vs. sham-operated group.

The effects on the learning and memory performances of the rats were evaluated using the Morris water maze test. Rats were trained for 6 consecutive days in the 2nd month after bilateral common carotid ligation. The results showed that the 2VO rats exhibited a significant memory deficit, as indicated by their longer escape latency (Figure 2). The chronic cerebral hypoperfusion induced by bilateral common carotid artery occlusion also caused working memory impairment as observed in the matching test compared to the sample test in Figure 1. The motor strength of the rats was tested using a prehensile traction test. The motor score showed no difference between groups, implying that no motor weakness was observed in the ischemic hypoperfusion model (Figure 2). However, there have been no reports about the other underlying mechanisms of neuronal damage protection by CTX treatment against, for example, white matter damage, oxidative stress, gliosis, inflammation, and apoptosis in chronic cerebral hypoperfusion models. Therefore, we further elucidated the effects of CTX treatment on these pathways in this 2VO chronic cerebral hypoperfusion model.

### 3.2. Effects of Ceftriaxone on White Matter Rarefaction

**Figure 3:**
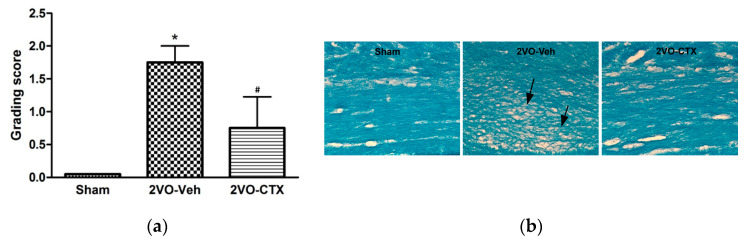
Special staining of Luxol fast blue (LFB) for the detection of white matter (WM) changes in the corpus callosum after ischemic induction and its protection by CTX treatment. Grading score of white matter changes in sham-operated rats, 2VO vehicle-treated rats, and 2VO CTX-treated rats (**a**). Representative images show white matter changes among groups (**b**). Nerve fiber disarrangement and vacuole formation were observed (arrowed). * *p* < 0.05 compared with sham-operated group; # *p* < 0.05 compared with 2VO-Veh-treated group. (*n* = 5).

White matter is vulnerable to ischemia, especially oligodendrocytes and myelinated axons. White matter damage has been demonstrated to be closely correlated with cognitive decline [23]. Therefore, we investigated the white matter changes in our chronic cerebral hypoperfusion and CTX-treated rats. White matter damage was significantly observed in the 2VO rats, and CTX treatment significantly exhibited beneficial effects by attenuating the white matter damage (Figure 3). Oxidative stress and inflammatory responses have been reported to play important roles in both gray and white matter damage and hypoperfusion-induced inflammatory events by the activation of microglia and astrocytes as well as the upregulated expression of inflammatory mediators such as interleukin 1 beta and TNF-alpha [24,25,26]. Thus, the effects of CTX treatment on oxidative stress, glial cells, and inflammatory mediator activation were further investigated in this study.

### 3.3. Effects of Ceftriaxone on Oxidative Stress

**Figure 4:**
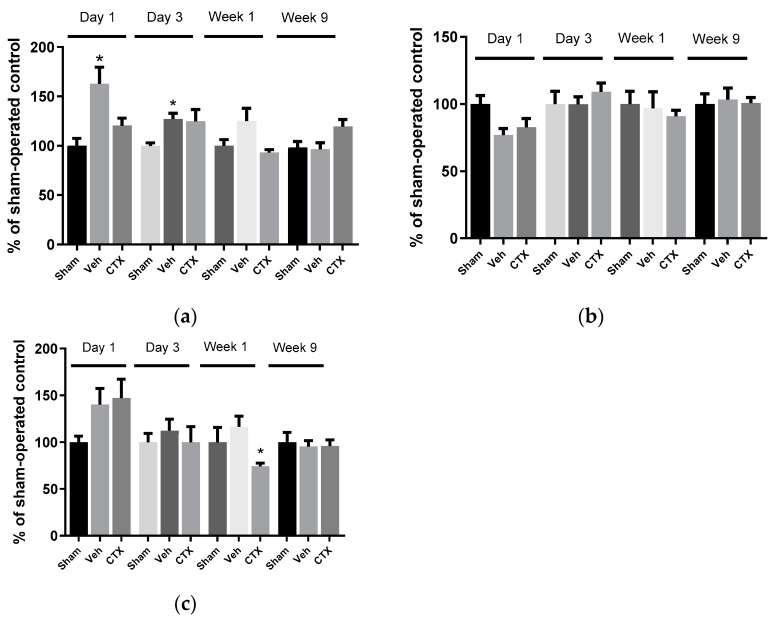
Malondialdehyde (MDA) level of rat brain tissues in the hippocampus (**a**), cortex (**b**), and striatum (**c**). MDA production significantly increased from ischemia and was attenuated by CTX treatment in the hippocampus. Data are expressed as mean ± SEM (*n* = 5). * *p* < 0.05 compared with sham-operated group.

To elucidate the role of oxidative stress in this model, the oxidative stress parameter of malondialdehyde (MDA) level was measured in tissues from several areas of the brain and at several timepoint after ischemia. The results showed that cerebral hypoperfusion significantly increased the MDA levels in 2VO rats in the hippocampal area on the first day and third day compared with the sham-operated group (sham). Elsewhere, no significant difference was observed in the MDA levels of the sham-operated and CTX-treated groups (CTX) (Figure 4). This indicated that CTX treatment could attenuate MDA production by inducing cerebral hypoperfusion in the hippocampus. However, a significant difference in the MDA levels between the groups was not observed in the cortex and striatum areas. Interestingly, ceftriaxone treatment significantly decreased the striatum MDA level in the first week compared with the sham-operated group. The results indicated that oxidative stress was involved in the chronic cerebral hypoperfusion, especially in the hippocampus area, and CTX treatment also attenuated this mechanism. This result relates to the finding that neuronal numbers in the hippocampus were decreased in chronic cerebral hypoperfusion model rats with memory impairment, and CTX treatment could attenuate neuronal damage as well as memory impairment.

### 3.4. Effects of Ceftriaxone on Gliosis

**Figure 5:**
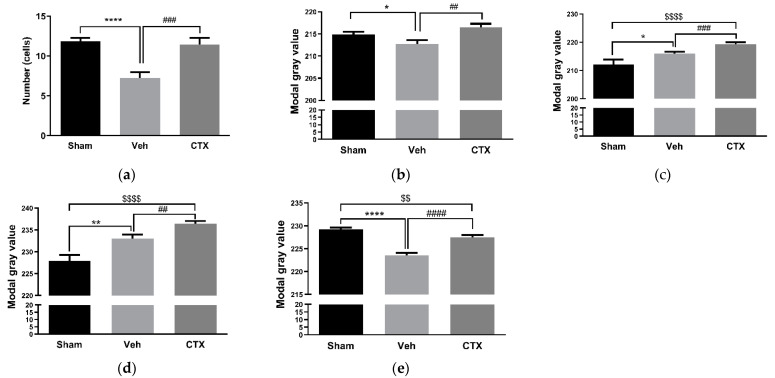
Immunohistochemistry of glial markers and glutamate transporters in the hippocampal sections. CTX treatment attenuated the reduction in the number of astrocytes and microglia by increasing the expression of GFAP (**a**) and OX42 (**b**), respectively. CTX treatment activated the expression of glutamate transporters in both glial cells and neuron cells by increasing the expression of EAAT1 (**c**), EAAT2 (**d**), and EAAT3 (**e**). *, **, and **** *p* < 0.05, 0.01, and 0.0001 Veh-treated group compared with sham-operated group. ##, ###, and #### *p* < 0.01, 0.001, and 0.0001 CTX-treated group compared with 2VO Veh-treated group. $$ and $$$$ *p* < 0.01 and 0.0001 CTX-treated group compared with sham-operated group (*n* = 5).

The effects of CTX treatment on gliosis were investigated using the immunohistochemical examination of the brain tissue 9 weeks after 2VO induction. GFAP and OX-42 were used as astrocyte and microglia activation markers, respectively. The results demonstrated the effect of 2VO on the reduction in the number of astrocytes and microglia, as observed through the significantly decreased expression of GFAP and OX-42 compared with the sham-operated group. CTX treatment could activate a number of astrocytes and microglia by significantly increasing the expression of GFAP and OX-42 compared with the 2VO-Veh-treated control, as shown in Figure 5a,b. To investigate the reactive response of glial cells, the expression of the glutamate transporters EAAT1 and EAAT2, which are primarily expressed in glial cells, was determined. EAAT1 and EAAT2 expression increased in both 2VO rats and CTX-treated rats. Interestingly, CTX treatment significantly increased EAAT1 and EAAT2 compared with the sham-operated control and 2VO-Veh-treated control groups (Figure 5c,d). This result indicated the reactive activation of glial cells and glutamate transporters’ expression after cerebral hypoperfusion. The expression of EAAT3 primarily in neuron cells was significantly decreased in both 2VO and CTX-treated groups compared with the sham group (Figure 5e) after cerebral hypoperfusion.

### 3.5. Effects of Ceftriaxone on Neuroinflammation

**Figure 6:**
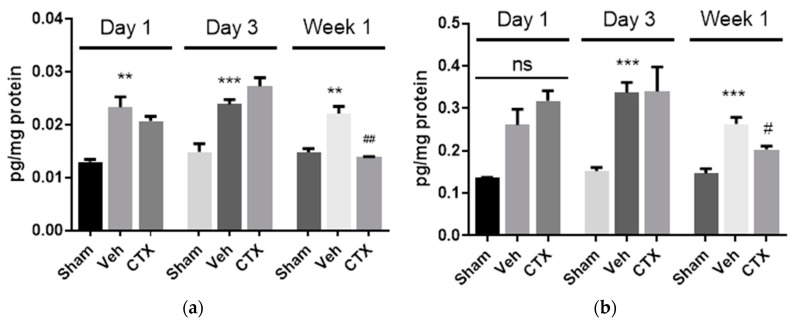
ELISA results of expression of inflammatory cytokines IL-1β (**a**) and TNFα (**b**) in the hippocampus. The expression of the inflammatory cytokines IL-1β (**a**) and TNFα (**b**) was increased in 2VO rats, and CTX-treated rats showed significantly decreased expression of IL-1β (**a**) and TNFα (**b**) in the first week after cerebral hypoperfusion. ** and *** *p* < 0.01 and 0.001 compared with sham-operated group; # and ## *p* < 0.05 and 0.01 compared with 2VO Veh-treated group (*n* = 5).

To investigate the effects of CTX on neuroinflammation, the expression of the pro-inflammatory mediator interleukin 1 beta (IL-1β) and tumor necrosis factor alpha (TNFα), which are pivotal in regulating immune responses following ischemia, was assessed. Hippocampal areas were investigated by ELISA. The expression of IL-1β was significantly increased in 2VO vehicle-treated rats at all time points. This indicated the effect of 2VO on the activation of IL-1β production (Figure 6a). Our results also demonstrated that the expression of IL-1β significantly decreased in the CTX-treated group compared with the 2VO control group in the first week after cerebral hypoperfusion induction. This indicated that CTX treatment reduced IL-1β expression and reduced the cell damage from cerebral hypoperfusion. Regarding the TNFα expression, there was no significant difference between the groups on the first day. However, the increased expression of TNFα was found in 2VO rats on the third day and first week after the 2VO experiment. In the first week after cerebral hypoperfusion, CTX treatment significantly attenuated TNFα expression compared to the 2VO vehicle-treated rats, similarly to the result of IL-1β expression.

## 4. Discussion

Chronic cerebral hypoperfusion induced by bilateral common carotid artery ligation caused learning, spatial memory, and working memory impairment, as observed in the matching test compared to the sample test. This agrees with a previously described 2VO model that induced neuronal damage or dysfunction due to the chronic cerebral hypoperfusion from bilateral common carotid artery occlusion. Ligation of the common carotid arteries caused a reduction in blood flow to approximately 40–65% of the normal CBF (Oligemia) [27,28,29]. Similarly to our results, it was reported that the learning and memory deficit in 2VO models is present without any motor deficits [30]. Neuronal injury or neuronal dysfunction by ischemia occurs as a result of the interaction between multiple complex pathways, including glutamate excitotoxicity, oxidative stress, gliosis, inflammation, and apoptosis [1]. The cognitive deficits in 2VO rats with chronic cerebral hypoperfusion are also correlated with oxidative stress and white matter damage [19,31]. Moreover, chronic cerebral hypoperfusion was also reported to induce free radical formation, glutamate excitotoxicity, neuronal death, and gliosis, which contribute to learning and memory impairments [30]. CTX has been reported to have neuroprotective and anti-ischemia effects by upregulating glutamate transporters and reducing glutamate excitotoxicity [10]. Moreover, post-treatment CTX could improve spatial learning and memory performance, as it increased the hippocampal neuronal number in the hippocampus in a chronic cerebral hypoperfusion model [14]. However, there have been no reports about the other underlying mechanisms. Therefore, we further elucidated the effects of CTX treatment on oxidative stress and inflammation pathways in this 2VO chronic cerebral hypoperfusion model. White matter is vulnerable to ischemia, especially oligodendrocytes and myelinated axons. White matter damage has been demonstrated to be closely correlated with cognitive decline [23]. White matter damage was significantly observed in the 2VO rats, and CTX treatment revealed significant beneficial effects. It has been reported that chronic cerebral hypoperfusion could induce white matter damage, along with the apoptosis of oligodendroglia [31]. Therefore, our result indicated that CTX plays a role in the attenuation of white matter and neuronal damage induced by chronic cerebral hypoperfusion. This result also strengthened an important point of the total brain protection obtained by the mitigation of gray and white matter damage from acute brain ischemia [26,32]. Oxidative stress and inflammatory responses have been reported to play important roles in both gray and white matter damage and hypoperfusion-induced inflammatory events by the activation of microglia and astrocytes as well as the upregulated expression of inflammatory mediators such as interleukin 1 beta and TNF-alpha [24,25,26].

MDA results showed that cerebral hypoperfusion was significantly increased in the hippocampal area a few days after ischemia. CTX treatment could decrease the MDA production, in agreement with the previous study, which showed a reduction in MDA levels in brain ischemia-reperfusion rats [33]. The hippocampal area plays an essential role in learning and memory and is rich in glutamatergic synapses and Ca^2+^-permeable AMPA receptor, which are vulnerable to excitotoxic damage. Since CTX treatment was found to upregulate glutamate transporter expression, it may contribute to a reduction in MDA by reducing excitotoxic damage in this area [34,35]. Moreover, there was no significant difference in the MDA levels of all the experimental groups in the ninth week. This indicated that oxidative stress pathways may only be involved in the early stages of cerebral ischemia. Several studies showed that the cerebral blood flow recovered to normal levels after the eighth week of permanent vessel ligation, in agreement with the normal MDA level found in the 9th week 2VO rats [27]. It has been reported that changes in the level of pro-oxidant and antioxidant enzymes in 2VO rats increased around the first week and declined after the second week and third month of chronic cerebral hypoperfusion, mainly due to their effects on cyclooxygenase-2 (COX-2) and endothelial nitric oxide synthase (eNOS) [36]

The effects of CTX treatment on gliosis were also investigated using immunohistochemistry. The results showed a reduction in the number of astrocytes and microglia compared with the sham-operated group. Astrocytes and microglia were stimulated by significantly increasing the expression of GFAP and OX-42. CTX treatment significantly increased EAAT1 and EAAT2 compared with the sham-operated control and 2VO-Veh-treated control groups. Our GFAP result contrasts with the previous study in traumatic brain injury which showed a reduction in GFAP expression in the lesion cortex in ceftriaxone treatment [37]. This result indicated the reactive activation of glial cells and glutamate transporters’ expression after cerebral hypoperfusion. The 2VO rat model has been reported to induce the activation of microglia that rapidly reacted with cerebral ischemia by synthesizing and releasing pro-inflammatory cytokines, reactive oxygen species, and eicosanoids [38,39]. CTX treatment may boost the reactive activation or prevent the death of astrocytes and microglia, protecting tissue from further damage. EAAT3 expression primarily in neuron cells was significantly decreased in both 2VO and CTX-treated groups compared with the sham group. This result correlates with several reports that found a reduction in the number of neuron cells after cerebral hypoperfusion [11,30]. Moreover, it has been reported that CTX treatment exerts neuroprotective effects by increasing neuron survival and glutamate transporter activity [11]. Altogether, these results could indicate that CTX treatment exerts neuroprotective effects by increasing reactive astrocytes and microglia, as well as through the upregulation of glutamate transporters’ expression, resulting in a reduction in glutamate excitotoxicity and an increase in neuron survival.

IL-1β expression was significantly increased in 2VO vehicle-treated rats at every time interval. It has been reported that a 2VO rat model could induce the release of pro-inflammatory cytokines and eicosanoids [38,39]. Their neurotoxic effects in ischemic stroke have been reported, and blocking IL-1β has been shown to reduce ischemic brain damage and cognitive impairment [24,40,41]. Our results also demonstrated that the expression of IL-1β was significantly decreased in the CTX-treated group compared with the 2VO control group in the first week after cerebral hypoperfusion induction. Our result is similar to the study of Lujia et al. regarding IL-1B in a focal ischemic rat model [42]. This indicated that CTX treatment reduced IL-1β expression and reduced the cell damage from cerebral hypoperfusion. TNFα increased on the third day and first week after 2VO induction. This is similar to previous reports [38,39]. This result indicated that CTX treatment inhibited the production of IL-1β and TNFα after the induction of cerebral ischemia. It has been reported that in the acute phase after the induction of cerebral ischemia, activated microglia are the predominant source of TNFα. Thereafter, blood-borne macrophages infiltrate and become the major sources of TNFα [43]. This supported our results explaining why we could not see a significant change at early observation times (first and third days).

In summary, the present study confirmed the CTX’s well known effects of improving learning and memory impairment in a chronic cerebral hypoperfusion rat model by reducing glutamate-excitotoxicity-induced neuronal damage [10,11,12,13]. Moreover, we also reported the underlying mechanism of CTX’s effect on chronic cerebral hypoperfusion, through the inhibition of glutamate-excitotoxicity and consequent mechanisms related to oxidative stress and inflammation, for the first time. Firstly, we demonstrated the effects that bilateral occlusion has on the common carotid artery in rats, including learning and memory deficits without any motor weakness, white matter damage, increased gliosis due to increased EAAT expression and decreased numbers of astrocytes and microglia, and increased oxidative stress markers (MDA) and pro-inflammatory cytokine (IL-1β and TNFα) levels. The major early production of pro-inflammatory cytokines and reactive oxygen species in this model may have been derived from the activation of microglia [31,38,43]. Early post-treatment with CTX led to functional protection from several underlying mechanisms involved in glutamate-excitotoxicity-induced learning and memory deficits. Treatment with CTX was found to attenuate white matter damage, MDA production, IL-1β but also TNFα production. Moreover, CTX treatment could increase the activation of astrocytes and microglia by increasing the cell numbers and expression of glutamate transporters EAAT1 and EAAT2 or by preventing glial cell death. Finally, CTX treatment was found to increase the expression of EAAT3, which is primarily expressed in neuronal cells; this agreed with the increased number of neuronal cells described in a previous study [14]. A treatment combining glutamate excitotoxicity, antioxidant, and anti-inflammation might be beneficial to protect neuronal damage from chronic cerebral hypoperfusion.

## 5. Conclusions

In conclusion, the present study highlighted the underlying mechanism of CTX treatment relative to the learning and memory deficits induced by glutamate excitotoxicity, and revealed the related pathophysiological mechanisms underlying glutamate-excitotoxicity-induced neuronal damage. Early post-treatment with CTX induced the upregulation of glutamate transporters’ expression, contributing to a reduction in glutamate excitotoxicity, oxidative stress, and pro-inflammatory cytokines production. This protective mechanism provides neuroprotection of neurons and tissue. Moreover, this early post-treatment appears to be beneficial for chronic cerebral hypoperfusion treatment. A combined treatment strategy might be beneficial to attenuate neuronal injury from chronic cerebral hypoperfusion, and could be a subject for further studies.

## Data Availability

Not applicable.
